# Arachidonic acid hyperpolarizes mesenchymal stromal cells from the human adipose tissue by stimulating TREK1 K^+^ channels

**DOI:** 10.1080/19336950.2019.1565251

**Published:** 2019-01-19

**Authors:** Michail V. Tarasov, Polina D. Kotova, Marina F. Bystrova, Natalia V. Kabanova, Veronika Yu. Sysoeva, Stanislav S. Kolesnikov

**Affiliations:** a Department of Molecular Cell Physiology, Institute of Cell Biophysics, Russian Academy of Sciences, Pushchino, Moscow Region, Russia; b Department of Biochemistry and Molecular Medicine, Faculty of Basic Medicine, Lomonosov Moscow State University, Moscow, Russia

**Keywords:** TREK-1 channel, patch clamp, arachidonic acid, mesenchymal stromal cells

## Abstract

The current knowledge of electrogenesis in mesenchymal stromal cells (MSCs) remains scarce. Earlier, we demonstrated that in MSCs from the human adipose tissue, transduction of certain agonists involved the phosphoinositide cascade. Its pivotal effector PLC generates DAG that can regulate ion channels directly or via its derivatives, including arachidonic acid (AA). Here we showed that AA strongly hyperpolarized MSCs by stimulating instantly activating, outwardly rectifying TEA-insensitive K^+^ channels. Among AA-regulated K^+^ channels, K2P channels from the TREK subfamily appeared to be an appropriate target. The expression of K2P channels in MSCs was verified by RT-PCR, which revealed TWIK-1, TREK-1, and TASK-5 transcripts. The TREK-1 inhibitor spadin antagonized the electrogenic action of AA, which was simulated by the channel activator BL 1249. This functional evidence suggested that TREK-1 channels mediated AA-dependent hyperpolarization of MSCs. Being mostly silent at rest, TREK-1 negligibly contributed to the “background” K^+^ current. The dramatic stimulation of TREK-1 channels by AA indicates their involvement in AA-dependent signaling in MSCs.

## Introduction

Mesenchymal stromal cells (MSCs) constitute a heterogeneous cell pool that includes immature cells, which replenish supportive and connective tissues owing to their capability of maintaining self-renewal and multipotent differentiation [1–3]. Intensive studies have been performed to reveal the mechanism underlying the capability of MSCs to migrate to sites of injury and to differentiate into functional cells, resulting in the regeneration of damaged tissues. The electrogenesis in MSCs in general and MSC specific ion channels in particular attracted much less attention. Meanwhile, existing evidence indicates that bioelectrical signaling not only regulates excitability but may also play an unexpectedly significant role in other cellular functions [4–6]. Reportedly, depolarization suppressed osteogenic and adipogenic differentiation of MSCs, while their hyperpolarization augmented osteogenic differentiation [5]. Furthermore, the depolarization of MSC-derived osteoblasts and adipocytes led to a marked down-regulation of bone and fat tissue markers despite the presence of differentiation-inducing chemical factors [7]. Thus, in this particular case, bioelectric signaling overrode biochemical signaling in the maintenance of the cell status. Several mechanisms can provide a link between membrane potential and intracellular biochemical processes. The common pathway is the voltage-dependent regulation of ion channels and electrogenic transporters, which mediate fluxes and therefore intracellular concentrations of metabolites and important regulatory ions, Ca^2+^ and H^+^ first of all. It also has been recognized that membrane voltage can affect cellular functions by modulating ligand binding to G-protein-coupled receptors (GPCRs) and coupling thereof to G-proteins as well as by regulating voltage-sensitive enzymes and altering the metabolism of phosphatidylinositol 4,5-bisphosphate (PIP_2_) [8].

Earlier, we found MSCs from the human adipose tissue to respond to a variety of GPCR ligands, including adrenergic and purinergic agonists, which mobilized cytosolic Ca^2+^ by stimulating phospholipase C (PLC) and IP_3_-dependent Ca^2+^ release [9,10]. By hydrolyzing PIP_2_, PLC affects the activity of PIP_2_-regulated channels [11], stimulates IP_3_ signaling, and produces the essential lipid messenger diacylglycerol (DAG) [12]. DAG, in turn, regulates a variety of ion channels either directly (e.g. TRPC) or indirectly, by stimulating PKC and/or originating downstream messengers, such as phosphatidic and arachidonic acids [12–14]. It is therefore not unlikely that the transduction of Ca^2+^-mobilizing agonists might be accompanied by a change in MSC polarization due to altered activity of ion channels regulated by PIP_2_, DAG, and downstream signaling molecules.

Reportedly, multiple extracellular signaling molecules may determine the fate of MSCs, including arachidonic acid (AA), which was particularly shown to promote MSC migration [15]. Since AA causes physiological effects through numerous cellular targets, including ion channels [13], we wondered whether AA might regulate the ion permeability of MSCs. Here we examined MSCs derived from the human adipose tissue and found that all cells assayed were hyperpolarized by AA. Our overall results suggested that the AA-dependent hyperpolarization of MSCs was primarily mediated by the TREK-1 channel, a member of the tandem pore class of K^+^ (K2P) channels.

## Materials and methods

### Cell isolation and culture

In this study, all procedures that involved human participants were performed in accordance with the ethical standards approved by the Bioethical Committee of Faculty of Basic Medicine at Lomonosov Moscow State University based on the 1964 Helsinki declaration and its later amendments. The study involved two healthy men of 29 and 45 years old, and informed consent was obtained from each participant.

MSCs were isolated from the subcutaneous fat tissue by enzymatic digestion as described previously [10]. Briefly, the adipose tissue was extensively washed with 2 volumes of Hank’s Balanced Salt Solution (HBSS) containing 5% antibiotic/antimycotic solution (10,000 units of penicillin, 10,000 μg of streptomycin, and 25 μg of amphotericin B per mL) (HyClone), fragmented, and then digested at 37°C for 1 h in the presence of collagenase (200 U/ml, Sigma-Aldrich) and dispase (10 U/ml, BD Biosciences). Enzymatic activity was neutralized by adding an equal volume of culture medium (Advance Stem basal medium for human undifferentiated MSCs containing 10% of Advance stem cell growth supplement (CGS), 1% antibiotic/antimycotic solution (HyClone)) and centrifuged at 200 g for 10 min. This led to the sedimentation of diverse cells, including MSCs, macrophages, lymphocytes, and erythrocytes, unlike adipocytes, which remained floating. After the removal of the supernatant, a lysis solution (154 mM NH_4_Cl, 10 mM KHCO_3_, and 0.1 mM EDTA) was added to a cell pellet to lyse erythrocytes, and the cell suspension was centrifuged at 200 g for 10 min. Sedimented cells were resuspended in the MSC culture medium and filtered through a 100 μm nylon cell strainer (BD Biosciences). After isolation and overnight preplating, the resulting cell population contained not only MSCs, which basically represented the most abundant subgroup, but also admixed macrophages and lymphocytes. The two last cell subgroups were dramatically depleted by culturing for a week in the MSC culture medium and humidified atmosphere (5% CO_2_) at 37°C. The resulting MSC population was maintained in the MSC culture medium at a sub-confluent level (~80% confluency) and passaged using HyQTase (HyClone). In experiments, MSCs of the second to sixth passages were used.

### RT-PCR analysis

Total RNA was isolated from a MSC sample (~10^6^ cells) by using the RNeasy Plus Mini Kit (Qiagen) and treated with DNase I (Ambion). By using Superscript III Reverse Transcriptase (Invitrogen) and random hexamer primers, cDNA was produced in a 20 µL reaction at 50°C for 1 h. PCR reactions were conducted with Ex Taq HS polymerase (Takara) and gene-specific primers that were designed to recognize all annotated transcript variants of the selected K2P channel genes and three genes encoding the cell-surface markers of the MSC phenotype, including CD73, CD90, and CD105 (Supplementary Materials, Table 1S). One primer in each pair spanned exons to avoid the amplification of gDNA.

Two sets of transcript-specific primers were designed to target transcript variants found for the human *KCNK2* (*TREK1*) gene (NM_001017424.2, NM_014217.3 and NM_001017425.2). The first primer pair (AGAGCCTCGGTTTGGAGTTC and GTGAGAATAAGTGACGCTGGC) specifically recognized transcript variant 1 (NM_001017424.2), spanning nucleotides 9–474 to yield a 466-bp product. The second primer pair (GCTCTCCCCACCTTGTAAAAC and AGAGCCTCGGTTTGGAGTTC) differentiated between transcript variants 2 and 3. It covered nucleotides 10–151 of variant 2 (NM_014217.3), with a 142-bp product, and nucleotides 10–275 of variant 3 (NM_001017425.2), with a 266-bp product. The primers were designed to anneal to sequences in different exons, so that products amplified from gDNA are much larger than products amplified from cDNA (80,878, 3078, and 3202 bp for variants 1, 2, and 3, correspondingly).

### Electrophysiology

Prior to electrophysiological experiments, MSCs were maintained for 12 h in the MSC culture medium without antibiotics. For isolation, cells cultured in a 1 ml socket were rinsed with the Versene solution (Sigma-Aldrich) and incubated in a HyQTase solution (HyClone) for 3–5 min. The enzymatic treatment was terminated by the MSC culture medium added to the socket. Next, MSCs were resuspended and put into a tube for storage in the MSC culture medium at 4°C for 6–8 h. When necessary, isolated cells were plated into a recording chamber of nearly 75 µl.

Ion currents were recorded with an Axopatch 200 B amplifier, and the data were collected with a Digidata 1440A interface and pClamp 10 software (Molecular Devices). When the perforated patch approach was used, the patch pipette was filled with (mM): 140 KCl, 1 MgCl_2_, 0.1 EGTA, 10 HEPES-KOH, pH 7.3, and 400 µg/ml Amphotericin B. The basic bath solution contained (mM): 135 NaCl, 5 KCl, 1 MgCl_2_, 2 CaCl_2_, and 10 HEPES-NaOH (NaOH), pH 7.4. The patch pipette contained 140 mM KCl, 1 MgCl_2_, 2 CaCl_2_, 10 HEPES-KOH, pH 7.3, and 10 mM TEA-Cl if cell-attached or inside-out recordings were performed, while the chamber was filled with the basic bath solution or intracellular solution (mM): 140 KCl (CsCl), 1 MgCl_2_, 1 Na_2_ATP, 1 EGTA+1 Ca^2+^ (~65 nM free Ca^2+^), 10 HEPES-KOH (CsOH), pH 7.4. At pH 5, 1 mM EGTA was substituted for 4 mM BAPTA in the intracellular solution that was not supplemented with Na_2_ATP. All chemicals were bath-applied for 1 s and rinsed within 3 s by using a gravity-driven perfusion system. The TREK-1 activity was characterized by the ***NP*_o_** value, where ***N*** is the number of channels active in a patch, and ***P*_o_** is the channel open probability. The values of ***NP*_o_** were determined by constructing all-points amplitude histograms of a patch current (0.2 pA bin width), taking single-channel conductance of 32 pS and using pCLAMP 10. Experiments were carried out at room temperature of 22–24°C. Arachidonic acid, ATP, buffers, salts, and TEA were from Sigma-Aldrich; BL1249 and spadin were from Tocris. Free Ca^2+^ concentration was evaluated using the Maxchelator program (http://maxchelator.stanford.edu). Averaged data were presented as means±s.d. The statistical analysis was performed using Sigma Plot 12.

## Results

**Figure 1.**
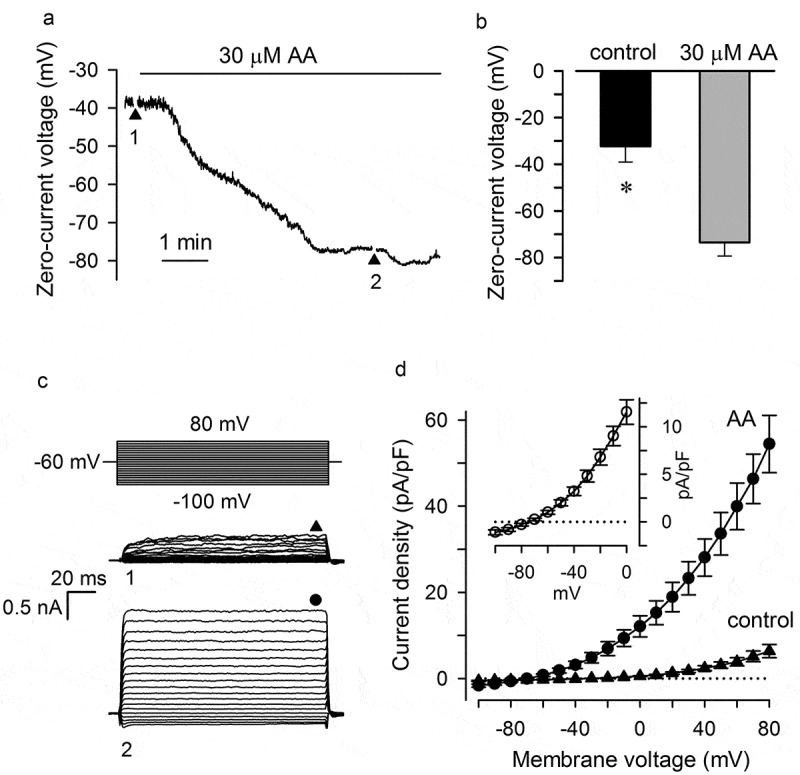
Hyperpolarization of MSCs by AA. (a) Evolution of membrane voltage after application of 30 µM AA. The zero-current clamp recording was interrupted at the moments 1 and 2 to generate I-V curves under the voltage clamp mode by polarizing a cell held at −60 mV by 150-ms voltage pulses from −100 to 80 mV. (b) Summary of membrane voltage in MSCs in control and at 30 µM AA. The data are presented as mean ± s.d. (n = 19). Here and below, the asterisk indicates significant difference (Student t-test at p < 0.05). (c) Families of integral currents elicited by voltage pulses (upper panel) in the same cell in control and with 30 µM AA in the bath. Currents were recorded at the moments 1 and 2 as indicated in (a). The steady-state currents were measured at the moments indicated by the symbols above the current traces. (d) Averaged (n = 19) current density versus membrane voltage in control and at 30 µM AA. Insert, the AA-gated current, which was determined as a difference between currents recorded in the presence and absence of AA, exhibited marked outward rectification and reversed at −75 mV on average. The perforated patch approach was employed with the pipette filled with 140 мМ KCl + 400 µg/ml amphotericin B; the bath solution contained 135 мМ NaCl + 5 мМ KCl.

To ascertain whether AA could serve as a physiologically relevant regulator of ion channels operating in MSCs, we first examined the effect of AA on the resting potential as a parameter sufficiently sensitive to a change in ion permeability at physiological voltages. Overall, we accomplished recordings of zero-current voltage from 27 MSCs, and each cell responded to AA (10–50 µM) with slowly developing hyperpolarization (Figure 1(a)). In most cases, cells (n = 19) were treated with 30 µM AA, which hyperpolarized cells from the resting potential of −41÷-25 mV to −82÷-65 mV (Figure 1(a,b)). Such strong hyperpolarization implied an increased activity of K^+^ channels as a primary mechanism, although some inhibition of Na^+^, cationic, or Cl^−^ channels by AA could not be excluded. As a rough probe for ionic selectivity of AA-sensitive channels, we generated current-voltage (I-V) curves just before the application of 30 µM AA (Figure 1(a), moment 1) and when the AA-dependent hyperpolarization reached a nearly steady-state level (Figure 1(a), moment 2). Because the input resistance markedly varied from cell to cell, the same current injection produced highly scattered voltage responses of different MSCs assayed using the current clamp mode. We therefore obtained I-V curves by switching the recording from the current to the voltage clamp mode for nearly 5 s to polarize a cell usually by 150-ms pulses (Figure 1(a), moments 1 and 2) or occasionally by a voltage ramp (1 mV/ms) (Figure 3(e)) from −100 to 80 mV. The representative families of voltage-gated (VG) integral currents, which were recorded in control (family 1) and in the presence of 30 µM AA (family 2), indicated that the fatty acid greatly enhanced almost instantly activating ion currents. When VG currents were normalized to cell capacitance, different cells became commensurable by current density (Figure 1(d)). The AA-sensitive current was defined as a difference between currents recorded from stimulated cells and in control. As illustrated in Figure 1(d) (insert), moderate outward rectification was characteristic of AA-activated currents that reversed between −85 and −65 mV, depending on a cell, or at −75 ± 7 mV on average (n = 19). This value is close to the Nernst potential of −85 mV calculated for the [140 mM K^+^]_in_/[5 mM K^+^]_out_ gradient at 22°C. It thus appeared that the electrogenic effect of AA was predominantly mediated by instantly activating and outwardly rectifying K^+^ channels.

**Figure 2.**
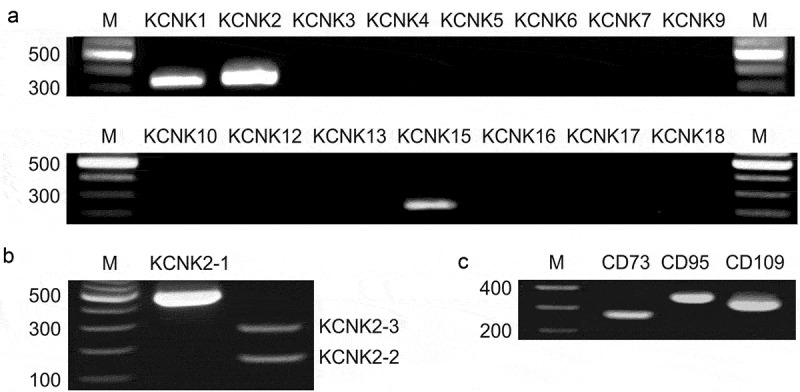
Expression analysis of K2P channels and the cell-surface markers of the MSC phenotype. (a) The detected amplicons of expected sizes (bp) correspond to transcripts for the *KCNK1* (334), *KCNK2* (361), and *KCNK*15 (211) genes. Products of expected sizes for other genes encoding K2P channels were not detected. (b) Expression of transcript variants of the *KCNK2 (TREK1)* gene in MSCs. RT-PCR analysis of MSCs with primers targeting transcript variant 1 (KCNK2-1) and primers that differentiate between transcript variants 2 (KCNK2-2) and 3 (KCNK2-3). The products of the expected sizes of 466, 142, and 266 bp were obtained for transcript variants 1, 2, and 3, correspondingly. (c) RT-PCR analysis of the expression of cell-surface markers CD73 (266 bp), CD90 (344 bp), and CD105 (317 bp). The molecular weight markers (M) were GeneRuler 100 bp DNA Ladder (Fermentas). The agarose gels (1.3%) were stained with ethidium bromide. No specific signals were detected in the no-RT controls.

Although known as an inhibitor of many K^+^ channels [13], AA also has been demonstrated to stimulate hEAG1 [16], Kir2.3 [17], and the TREK subfamily of K2P channels, including TREK-1, TREK-2, and TRAAK [18]. Based on their voltage dependence and activation kinetics (Figure 1(c,d), AA-gated K^+^ currents identified in MSCs could hardly be attributed to the activity of hEAG1 or Kir2.3. To elucidate whether K^+^ channels of the TREK type were functionally expressed in MSCs, we first searched for transcripts related to some of 15 human genes encoding K2P channels, namely, *KCNK1-KCNK7, KCNK9, KCNK10, KCNK12, KCNK13, KCNK15-KCNK18*. By using the conventional RT-PCR and gene-specific primers, we detected products of the expected sizes for *KCNK1* (TWIK-1), *KCNK2* (TREK-1), and *KCNK15* (TASK-5) in all analyzed RNA preparations (n = 4), each being obtained from a separate MSC colony (~10^6^ cells). Transcripts for the other K2P genes were not detected (Figure 2(a)). Thus, among K2P channels, only TWIK-1, TREK-1, and TASK-5 subtypes were identified in MSCs, and by biophysical features, solely TREK-1 was suitable for mediating AA-gated K^+^ currents (Figure 1(c,d)). Three transcript variants encoding different isoforms have been found for the human TREK1 gene (NM_001017424.2, NM_014217.3, and NM_001017425.2; NCBI database). The longest variant 1 differs in the 5ʹ UTR and beginning of the coding region compared to variants 2 and 3, while the first exon of the variant 2 is shorter compared to the variant 3. The RT-PCR analysis of MSCs with transcript-specific primers revealed mRNAs for all three transcript variants of the *TREK1* gene (Figure 2(b)).

**Figure 3.**
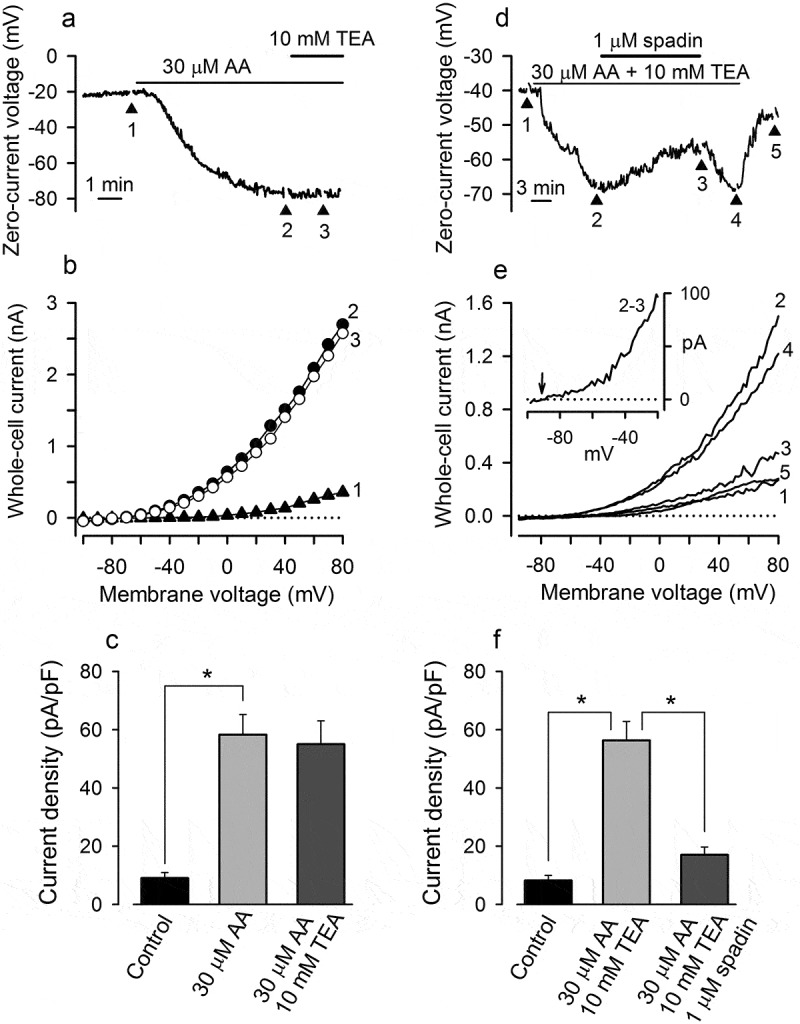
AA-gated channels are insensitive to TEA but blockable with spadin. (a, b) 10 mM TEA did not reverse MSC hyperpolarization elicited by 30 µM AA and an associated increase in the membrane conductance A. The I-V curves 1–3 in (B) were generated at the corresponding moments in (A) as described in Figure1. (c) Averaged (7 cells) current density at 80 mV in control and with 30 µM AA or with 30 µM AA +10 mM TEA in the bath. There is no significant difference between averaged currents recorded in the presence of 30 µM AA or 30 µM AA+10 mM TEA (p < 0.05); the paired asterisks indicate significant difference compared to control at p < 0.01. (d, e) Spadin (1 µM) partly reversed MSC hyperpolarization induced by 30 µM AA and strongly suppressed AA-gated conductance. The I-V curves in (e) were generated by voltage ramps (1 mV/ms) in the corresponding moments indicated in (D). Insert, the spadin blockable AA-gated current was determined as a difference between currents recorded with 30 µM AA (curve 2) and 30 µM AA + 1 µM spadin (curve 3) in the bath. (f) Current density at 80 mV in control and in the presence of 30 µM AA+10 mM TEA or 30 µM AA+10 mM TEA + 1 µM spadin (5 cell). In all cases, the recording conditions were as in Figure1.

The TREK-1 channel displays specific pharmacological properties. In particular, it is poorly sensitive, as the whole K2P family, to classical blockers of K^+^ channels, including TEA [18], but is specifically blockable by spadin [19]. Thus, the relative sensitivity of AA-gated currents to spadin and TEA could allow for evaluating the contribution of TREK-1. It turned out that 10 mM TEA negligibly affected both hyperpolarization elicited by 30 µM AA (7 cells) (Figure 3(a)) and I-V curves generated during voltage evolution (Figure 3(b), curves 2 and 3; Figure 3(c)), indicating an imperceptible sensitivity of AA-gated channels to TEA. On the other hand, 1 μM spadin partly reversed MSC hyperpolarization produced by 30 μM AA in the presence of 10 mM TEA (Figure 3(d)), the effect being accompanied by a marked decrease in the AA-dependent conductance (Figure 3(e), curves 2 and 3; Figure 3(f)) (5 cells). The AA-gated current reversed between −85 and −77 mV (Figure 3(e), insert), implicating TEA-insensitive, spadin-blockable K^+^ channels, presumably of the TREK-1 type.

**Figure 4.**
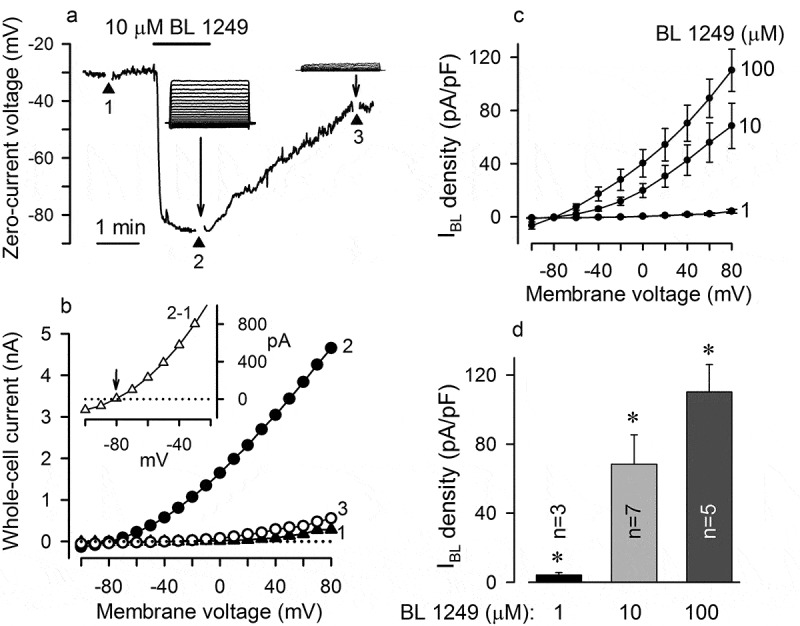
BL 1249 hyperpolarizes MSCs. (a) Evolution of MSC voltage in control and after application of 10 µM BL 1249. Inserts, BL 1249 reversibly stimulated an outwardly rectifying current, which was similar to the AA-gated current. (b) I-V curves generated at the moments 1–3 in (A). The BL 1249-gated current (insert), which was determined as a difference between currents recorded in control and in the presence of 10 µM BL 1249, reversed at −81 mV. (c) Averaged density of the BL 1249-gated current versus membrane voltages at the BL 1249 concentrations of 1 (3 cells), 10 (7 cells), and 100 (5 cells) µM. (d) Summary of BL 1249-gated current density at 80 mV with 1, 10, and 100 µM BL 1249 in the bath. The recording conditions were as in Figure1.

In search for additional evidence, we examined the effects of BL 1249, an activator of the TREK-1 channel [20], and found this compound to elicit a dramatic and reversible hyperpolarization of MSCs (Figure 4(a)). The BL 1249-dependent negative shift of membrane voltage was associated with an increase in instantly activated currents (Figure 4 (a), inserts), which exhibited the outward rectification and reversed at −79 ± 4 mV (9 cells) (Figure 4(b)). Acting in a dose-dependent manner, BL 1249 was weakly effective at 1 μM but markedly increased K^+^ conductance at 10 μM and higher concentrations (Figure 4(c,d)). Although 100 μM BL 1249 did not elicit the maximal effect, the pipette/cell tight contact was usually lost at higher concentrations, precluding us from generating an accurate dose dependence for the BL 1249-activated current. It is noteworthy that the stimulatory effect of BL 1249 was reversible at moderate doses and observed in all treated cells (n = 12) (Figure 4(a,b)). The similarity of the electrogenic effects of BL 1249 and AA together with the inhibitory action of spadin (Figure 4(d,e)) pointed at TREK-1 channels to mediate the AA-dependent hyperpolarization associated with an increase in K^+^ permeability of the MSC plasma membrane (Figure 1).

**Figure 5.**
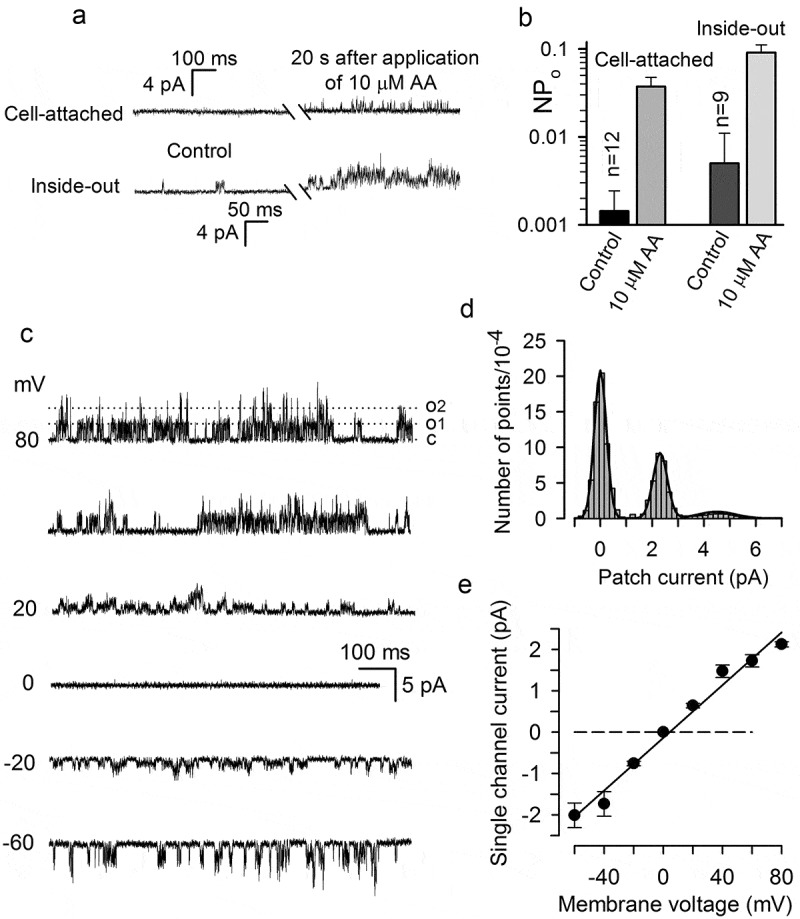
Minor activity of AA-gated K^+^ channels in resting MSCs. (a) Single channel activity in cell-attached (upper panel) and inside-out (bottom panel) patches in control and with 10 µM AA in the bath. The recordings were performed from two different cells under voltage clamp with −60 mV in the pipette containing 140 mM KCl+10 mM TEA. The bath contained 135 mM NaCl+5 mM KCl or 140 mM KCl in the case of cell-attached or inside-out recordings, respectively. (b) Summary of the activity of AA-gated K^+^ channels in cell-attached (upper panel) and inside-out (bottom panel) patches with −60 mV in the pipette. The paired asterisks indicate significant difference compared to control at p < 0.01. (c) Representative (n = 7) single-channel activity at indicated voltages in an inside-out patch stimulated by 5 µM AA. (d) Amplitude histogram of the patch current at 80 mV shown in (C). This histogram was fitted with the expression $F(i)=\overset{3}{\underset{k=1}{\sum }}a_{k}\exp(-((i-i_{k})/\sigma_{k}{)}^{2})$ that is the sum of three Gaussians, at *a*_1_-20.8*10^4^, *a*_2_ = 9.2*10^4^, *a*_3_ = 9.2*10^4^; *i*_1_ = 0, *i*_2_ = 2.35, i_3_ = 4.5 pA; *σ*_1_ = 0.35, *σ*_2_ = 0.39; *σ*_3_ = 0.94 pA. The difference *i*_2_-*i*_1_ = 2.35 pA was taken as a measure of the single-channel current at 80 mV. (e) Summary of single-channel currents at different voltages. The data are presented as mean ± s.d. (n = 7). The single-channel current versus membrane voltage allowed for the linear approximation with the slope of 31.9 ± 1.9 pS on average.

In a number of experiments, we examined the action of AA on membrane patches, keeping in mind that AA could stimulate TREK-1 directly [21]. With the patch pipette filled with 140 mM KCl + 10 mM TEA, we obtained 12 sufficiently stable cell-attached patches, wherein the single-channel activity was subtle in control (NP_0_ = 0.0014 ± 0.001) at −60 mV in the recording pipette. It should be mentioned that in an inside-out patch, the membrane potential was set just by command voltage. In contrast, transmembrane voltage in a cell-attached patch was an algebraic sum of command voltage, 60 mV in the given case (−60 mV in the pipette), and the resting potential that varied between −20 and −40 mV from cell to cell (Figure 1(b)). The exposure of a cell to 10 µM AA initiated single-channel activity in a cell-attached patch with NP_0_ = 0.037 ± 0.012 on average (n = 12) (Figure 5(a), upper panel; Figure 5(b)). When a patch fragment was excited from a cell in the presence of AA, gigaseal commonly disappeared. Therefore, the activity of AA-gated channels in inside-out patches was analyzed in independent experiments, wherein excised patches were obtained first, and then those were stimulated by micromolar AA from the cytosolic side. Overall, 30 inside-out patches were obtained, and all contained AA-stimulated channels the activity of which was low in control but markedly strengthened by 10–20 µM AA (Figure 5(a), bottom panel). For nine sufficiently stable inside-out patches, which contained only few AA-gated K^+^ channels, we were capable of quantifying single-channel activity. It was particularly found that, at 60 mV, averaged NP_0_ was 0.005 ± 0.003 in control but rose to 0.09 ± 0.02 upon stimulation with 10 µM AA (Figure 5(b)). In addition, we assayed AA-stimulated patch currents at varied voltage (Figure 5(c)) and designed their amplitude histograms (7 patches) (e.g. Figure 5(d)) to evaluate the conductance of individual AA-gated channels. These experiments showed that from patch to patch, AA activated K^+^ channels that were characterized by similar flickering-burst kinetics (Figure 5(c)) and apparently the same single-channel conductance of nearly 32 pS (Figure 5(e)).

**Figure 6.**
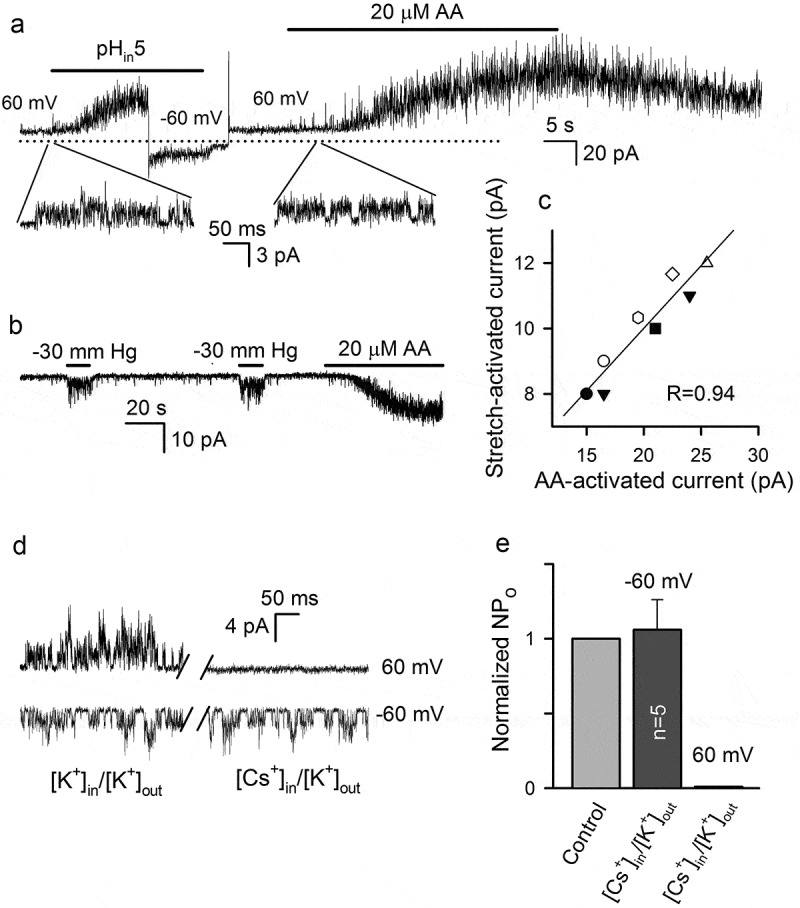
Effects of different factors on the activity of AA-gated K^+^ channels. (a) Representative (n = 8) current recording from an inside-out patch stimulated by acidification of bath solution (140 mM KCl, pH 5) and by 20 µM AA at pH 7.4 at membrane voltages varied as indicated. The recording pipette contained 140 mM KCl and 10 mM TEA-Cl. The below inserts represent single-channel activity at extended time scale. The zero-current level is indicated by the dotted line. (b) Representative (n = 8) current at −60 mV recorded from an inside-out patch that was held at −2 mm Hg and sequentially stimulated by suction pressure pulses (−30 mm Hg) and by 20 µM AA. The recording conditions were as in (A). (c) Correlation between stretch-activated and AA-gated currents. The straight line corresponds to linear regression with the correlation coefficient R = 0.94. The stretch-activated current was determined as a difference between averaged values of control and stimulated patch currents, which were recorded for 2 s just before and after application of suction pressure. The AA-gated current was determined as a difference between averaged values of patch currents recorded before stimulation and after AA application when a patch current attained a steady-state level. (d) Substitution of 140 mM KCl for 140 mM CsCl in the bath inhibited the outward but not inward single-channel current stimulated by 10 µM AA from inside. (e) Summary of activity (NP_o_) of AA-gated channels in the symmetric K^+^ saline (control) and under the [K+]_out_/[Cs^+^]_in_ gradient at −60 and 60 mV. In each case, the value of NP_o_ in control was taken as 1.

TREK-1 is known as a polymodal K^+^ channel that responds to a variety of physical and chemical cues, including acidosis, membrane stretch, temperature, and GPCR-mediated intracellular signals [18,22–24]. We therefore wondered if some of these features were also characteristic of AA-gated TREK-1-like channels operating in MSCs (Figures 1, 3–5). It turned out that the acidification of internal K^+^ saline to pH 5 stimulated slowly developing currents in inside-out patches (n = 8) that were reminiscent of AA-gated currents (Figure 6(a)). The switch of membrane voltage from 60 to −60 mV markedly decreased the value of the H^+^-activated current, indicating one to be outwardly rectifying (Figure 6(a)). Given that both acidification and 20 µM AA initiated an activity of K^+^ channels of nearly 32 pS with flickering-burst kinetics (Figure 6(a), inserts), presumably the same K^+^ channels responded to both stimuli.

In some experiments, we tried to evaluate the stretch sensitivity of AA-gated channels and found first that suction pressure (20–30 mm Hg) increased K^+^ conductance in none-stimulated inside-out patches (n = 14). Unfortunately, we failed to directly demonstrate the mechanosensitivity of AA-gated K^+^ channels because micromolar AA destabilized inside-out preparations in that gigaseal quickly collapsed when sufficient suction pressure was applied. Thus, instead of direct demonstration, we aimed at obtaining correlational evidence by stimulating inside-out patches mechanically and then by AA (Figure 6(b)). In all these cases (n = 8), suction pressure (30 mm Hg) induced a quick and reversible increase in patch currents, which also were strengthened by 20 µM AA (Figure 6(b)). By graphing stretch-activated current versus AA-activated current for each patch (Figure 6(c)), we revealed a strong interdependence between current values, which was characterized by the correlation coefficient of R = 0.94. This finding argued for that both membrane stretch and AA stimulated the same K^+^ channels. Yet, Cs^+^ acts on TREK-1 as an impermeable ion rather than a blocker [25]. Consistently, when K^+^ was replaced with Cs^+^ in the internal solution bathing an inside-out patch (n = 5), the outward single-channel current was completely inhibited, while the inward component remained virtually unchanged compared to control (Figure 6(e)).

## Discussion

Arachidonic acid (AA) and its derivatives are ubiquitous regulators of cellular functions, including electrogenesis [26–28]. Increasing body of evidence implicates AA-dependent signaling in governing the physiological status of stem cells in general and MSCs in particular [28]. Reportedly, AA is an essential component of a culture medium capable of maintaining MSC proliferation [29]. This fatty acid inhibits osteoblastogenesis and induces adipogenesis of MSCs [30] and regulates MSC migration by involving the fatty acid receptor GPR40 [15]. Derived from AA and acting via EP GPCR receptors, prostaglandin E_2_ is secreted by MSCs as an autocrine factor involved in their self-renewal [31].

Here we studied electrogenic effects of AA on MSCs from the human adipose tissue and found this fatty acid to strongly hyperpolarize all cells tested (Figure 1(a,b)) by increasing their permeability to K^+^ ions (Figure 1(c,d)). Among likely mechanisms, we particularly considered the possibility that AA could activate Ca^2+^ entry channels [32], thereby elevating cytosolic Ca^2+^ and stimulating Ca^2+^-gated K^+^ channels, which have been identified in MSCs from different sources, including the human adipose tissue [33,34]. However, these Ca^2+^-gated K^+^ channels were blocked by TEA [33,34], and therefore their contribution to the TEA-independent electrogenic effects of AA on MSCs (Figure 3(a,b)) might not be essential. This inference was supported by the observation that 10–30 µM AA never elevated cytosolic Ca^2+^ to the level (Figure 1S) required for sufficient MSC hyperpolarization associated with the activation of Ca^2+^-gated K^+^ channels [34]. On the other hand, AA-gated K^+^ channels identified by us in MSCs were most closely related to the TREK-1 type by biophysical and pharmacological properties [18,22–24,35], including their inhibition by spadin (Figure 3(d,e)) and stimulation by BL 1249 (Figure 4), intracellular acidosis (Figure 6(a)), and membrane stretch (Figure 6(b,c)).

Initially, mammalian TREK-1 has been described as K^+^ channels of 90–130 pS in 140–155 mM symmetric K^+^ saline [36–38]. Several alternatively spliced variants of human TREK-1 have been identified and shown to alter the properties of a basal TREK-1 related current when co-expressed with the full-length TREK-1 [39]. It also has been shown that alternative translation initiation generates full-length and truncated proteins from a single TREK-1 transcript. In a heterologous system, each TREK-1 subunit is capable of forming channels with specific biophysical and pharmacological properties [40]. In particular, the full and short TREK-1 isoforms from the rat were characterized by the single channel conductance of 53 pS and 93 pS in symmetric saline (150 mM KCl) at −60 mV, respectively [40]. Moreover, K2P subunits form not only homodimeric channels [41] but also certain heterodimers [42]. Thus, both alternative splicing and translation extend a set of K2P channels, particularly TREK-1, endowed with different properties, including single-channel conductance. Here we found MSCs to express transcript variants encoding three distinct TREK-1 isoforms. This molecular diversity may account for the inconsistency in values of the single-channel conductance reported for different TREK-1 isoforms and the estimated value of 32 pS obtained by us for TREK-1-like channels operating in MSCs (Figure 5(c-e)).

**Figure 7.**
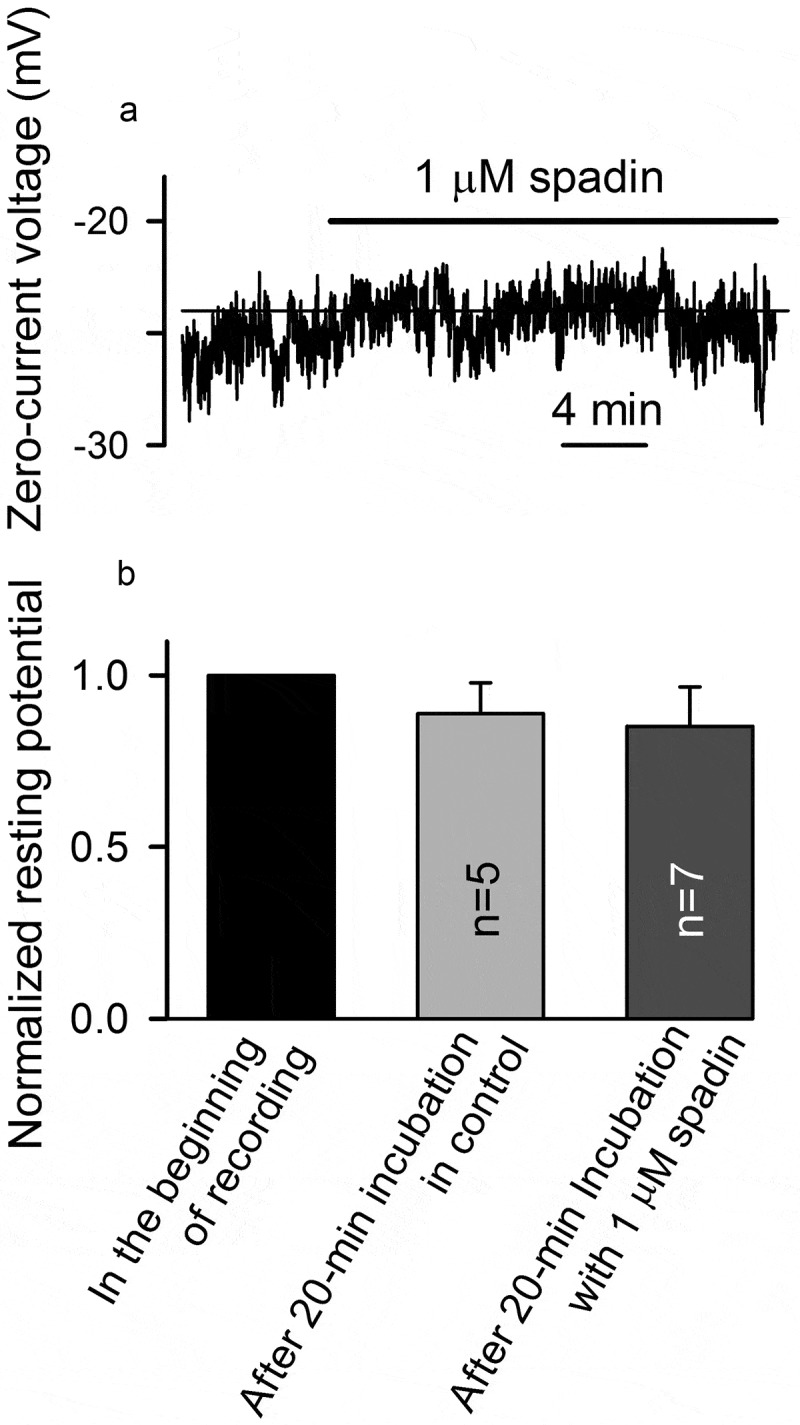
TREK-1 channels contribute negligibly to the resting potential in MSCs. (a) Resting potential was insignificantly sensitive to 1 µM spadin. The recording conditions were as in Figure1. (b) Summary of spadin effects on the resting potential in MSCs. Two separated recordings were performed as membrane voltage recorded in non-stimulated cells varied with time to certain extent, while spadin effects developed slowly (Figure2(d)). In 5 cells assayed in control, membrane voltage first was measured in the very beginning of the recording (taken as 1) and then 20 min after incubation in the bath solution. MSCs treated with 1 µM spadin for 20 min (n = 7) were statistically undistinguishable (p < 0.05) from control cells by membrane voltage.

It is widely accepted that K2P channels, including TREK-1, generate a “background” K^+^ current, thereby setting the resting potential and providing a pathway for the regulation of excitability of neurons, muscles, and secretory cells [18]. In our experiments with non-stimulated MSCs, TREK-1-like channels exhibited a surprisingly low activity (NP_0_ = 0.0014 ± 0.001) in cell-attached patches (Figure 5(a), upper panel; Figure 5(b)), which was markedly increased in excised patches (Figure 5(a), bottom panel; Figure 5(b)). Consistently, spadin negligibly affected membrane voltage in non-stimulated MSCs (Figure 7), indicating a subtle contribution of TREK-1-like channels to the resting potential. To all appearance, a currently non-identified mechanism down-regulated TREK-1-like channels in resting MSCs. Perhaps, these channels serve mainly as a downstream effector in agonist signaling, given that AA markedly increased the activity of TREK-1-like channels in MSCs (Figure 4(a,b)) and strongly hyperpolarized these cells (Figure 1(a,b)). The true molecular nature of TREK-1-like channels and their functional role in MSC physiology remain to be elucidated.
