# Supplementary material for: Arachidonic acid hyperpolarizes mesenchymal stromal cells from the human adipose tissue by stimulating TREK1 K+ channels
Source: Channels (Austin). 2019 Jan 19;13(1):36–47. doi: 10.1080/19336950.2019.1565251 (PMC6380217; doi:10.1080/19336950.2019.1565251)
Supplement: Supplemental Material [file kchl-13-01-1565251-s001.pdf]

Supplementary Materials

1. *RT-PCR analysis of expression of K2P channels in MSCs*

Table 1S. Sequences of gene-specific primers

**Target gene**	**5′-sequence-3′** (Forward/Reversed)	**Product size (bp)**
KCNK1 (TWIK1)	GATTGGGATCACGTGTTACC<br>TTAAGCCTCTTCCTTGCACC	334
KCNK2 (TREK1)	GTGGAAGATACGTTTATTAAGTGG<br>CTCTGAACTCTCCCACCTC	361
KCNK3 (TASK1)	TCATCACCACCATCGGCTAC<br>GTAGTAGGCCTGGAAGAAG	305
KCNK4 (TRAAK)	AAGCCATCTTCTTGAAGTGG<br>ACTACTCGCAGCCAGTTC	319
KCNK5 (TASK2)	ACCGTCATTACCACCATTGG<br>TAG TTG GCG CTG GGG TTC	377
KCNK6 (TWIK2)	TGTCTTGGCTGAGCATGCGT<br>GCCCAGGAAGAGGTAGACTG	275
KCNK7	CATCCTCACCACCACAGG<br>GTCCTAGAAGCAAGTAACCAAG	430, 530, 630, or 730 depending on splice variant
KCNK9	ACGGTCATCACCACCATAGG<br>AAGCTCCACTCCTCACAC	293
KCNK10 (TREK2)	AACTGTCATTACGACCATAGGG<br>GCCTTGATTTCACCCACCTC	528
KCNK12	GGTGTCAACCATAGGTTTCG<br>ATGAGCAGCACGTGGTAC	279
KCNK13	TCATCGTGCTCTACCTGC<br>GTTGTCATCCCAAACCCTATG	274
KCNK15 (TASK5)	CGTCATCACTACCATCGAGTAC<br>CAGGTTCTCCGTGGACAC	211
KCNK16	ACAGTCGTCACTACCATAGG<br>CTCCACATGGCTGAAGAC	276
KCNK17	ACTCGCTGATCCGGGATG<br>CCCAGTGGTTTACTCCCTG	264
KCNK18	TTCAGCACCGTGGGCTATG<br>CGTGAAGGACATGTGCCAAG	320
CD73	AAGACATGACTCTGGTGACC<br>CTGTCACAAAGCCAGGTCC	266
CD90	GTCACAGTGCTCAGAGAC<br>TACAAAAAGACAGCCAGAGG	344
CD105	AGCAGAGCTTTGTGCAGGTC<br>GCTGATGATGTTCAAGCGCATG	317

2. Effects of arachidonic acid on cytosolic Ca^{2+} in MSCs.

In a number of experiments, the responsiveness of MSCs to AA and ATP, an effective Ca^{2+} mobilizer, was assayed with Ca^{2+} imaging. As illustrated in Fig.1S, 10 μM AA affected cytosolic Ca^{2+} inessentially in all cells assayed (n=76), while purinergic MSCs (9 cells) responded to 3 μM ATP with marked Ca^{2+} transients sufficient to activate Ca^{2+}-gated K^+ channels (Tarasov et al. Pflugers Arch. 2017; 469: 349-362).

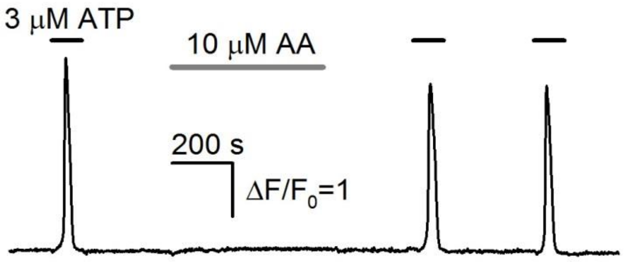

Figure 1S. Representative monitoring of intracellular Ca^{2+} in MSC stimulated by 3 μM ATP and 10 μM AA.

Method. For Ca^{2+} imaging, cells were plated onto a photometric chamber of nearly 150 μl volume. This chamber was a disposable coverslip (Menzel-Glaser) with an attached ellipsoidal resin wall. The chamber bottom was coated with Cell-Tak (BD Biosciences), enabling strong cell adhesion. Attached cells were then loaded with the Ca^{2+} indicator Fluo-4 for 20 min at room temperature (23–25°C) by adding Fluo-4AM (4 μM) or Fluo-4AM (4 μM) + NP-EGTA-AM (4 μM) and Pluronic (0.02%) (all from Molecular Probes) to a bath solution. Loaded cells were rinsed with the bath solution three times and kept at 4°C for 1 hour prior to recordings. Generally, the incubation of MSCs at low temperature stabilized intracellular Ca^{2+} and decreased a fraction of spontaneously oscillating cells.

Cells loaded with Fluo-4 were imaged using a fluorescent microscope Axiovert 135 equipped with an objective Plan-Neofluar 20×/0.75 (Zeiss) and ECCD camera LucaR (Andor Technology). Fluo-4 fluorescence was excited at 480±5 nm using LED controlled by a computer. Fluo-4 emission was collected at 535±20 nm. Serial fluorescent images were captured every second and analyzed using the Imaging Workbench 6 software (INDEC). Deviations of cytosolic Ca^{2+} from the resting level were quantified by a relative change in the intensity of Fluo-4 fluorescence ($\Delta F/F_0$) recorded from an individual cell.
